# Metabolic Syndrome, Obesity, and Gastrointestinal Cancer

**DOI:** 10.1155/2012/483623

**Published:** 2012-12-11

**Authors:** Shintaro Fujihara, Hirohito Mori, Hideki Kobara, Noriko Nishiyama, Mitsuyoshi Kobayashi, Makoto Oryu, Tsutomu Masaki

**Affiliations:** Department of Gastroenterology and Neurology, Faculty of Medicine, Kagawa University, 1750-1 Ikenobe, Miki-cho, Kita-gun, Takamatsu, Kagawa 761-0793, Japan

## Abstract

Metabolic syndrome is a cluster of metabolic abnormalities and is defined as the presence of three or more of the following factors: increased waist circumference, elevated triglycerides, low high-density lipoprotein cholesterol, high blood pressure, and high fasting glucose. Obesity, which is accompanied by metabolic dysregulation often manifested in the metabolic syndrome, is an established risk factor for many cancers. Adipose tissue, particularly visceral fat, is an important metabolic tissue as it secretes systemic factors that alter the immunologic, metabolic, and endocrine milieu and also promotes insulin resistance. Within the growth-promoting, proinflammatory environment of the obese state, cross-talk between macrophages, adipocytes, and epithelial cells occurs via obesity-associated hormones, adipocytokines, and other mediators that may enhance cancer risk and progression. This paper synthesizes the evidence on key molecular mechanisms underlying the obesity-cancer link.

## 1. Introduction

The clustering of atherosclerotic risk factors that identify metabolic syndrome was first recognized in 1983 [1]. In 1988, Reaven [2] introduced the term *syndrome X*, in which insulin resistance (IR) was the common denominator. In 1998, the World Health Organization recommended a unifying definition and chose the term metabolic syndrome [3], primarily because the available data did not support IR as the cause of all of the components of this syndrome [4].

Metabolic syndrome is now recognized as a cluster of metabolic abnormalities and is defined as the presence of three or more of the following factors: abdominal obesity (increased waist circumference), elevated triglycerides, low high-density lipoprotein (HDL) cholesterol, high blood pressure, and high fasting glucose [5].

IR, along with its associated hyperinsulinemia and hyperglycemia, and adipocyte cytokines (adipokines) may lead to vascular endothelial dysfunction, an abnormal lipid profile, hypertension, and vascular inflammation, all of which promote the development of atherosclerotic cardiovascular disease (CVD) [2, 6–8].

The metabolic syndrome involves a proinflammatory, prothrombotic state and is associated with elevated levels of C-reactive protein [9–12], interleukin (IL)-6 [12], and plasminogen activator inhibitor (PAI)-1 [13]. These inflammatory and prothrombotic markers are also associated with an increased risk for subsequent CVD and type 2 diabetes [11, 12].

In addition to the mechanical effects of obesity, such as an increase in intra-abdominal pressure caused by excess adipose tissue, adipose tissue secretes various adipokines, such as tumor necrosis factor (TNF), IL-6, leptin, and insulin-like growth factor-1 (IGF-1), which are considered to have pathogenic effects in relation to gastrointestinal cancer.

The classification of obesity for epidemiological purposes defines overweight as body mass index (BMI) >25 kg/m^2^ and obesity as BMI >30 kg/m^2^ [14]. Fat is principally deposited in two compartments—subcutaneous and central depots. It is thought that central fat (i.e., visceral fat) is more metabolically active than peripheral subcutaneous fat [15, 16].

In the Asian Cohort Consortium, the East Asian, Indian, and Bangladeshi populations with a BMI <20.1 kg/m^2^ had significantly higher mortality compared with subjects with a BMI of 22.6–25 kg/m^2^ [17]. Mortality was lowest among men and women with a BMI in the range of 22.6–27.5 kg/m^2^. 

Omental adipose tissue, also referred to as visceral adipose tissue, can exert systemic effects that are putatively involved in cancer biology [18]. Metabolic syndrome is defined based on a cluster of abnormal clinical factors that have been associated with the development of CVD morbidity [19] and type 2 diabetes mellitus [20]. The definitions used in this paper are summarized in Table 1 [3, 5, 21–23].

Overall, 14% of all cancer deaths in men and 20% of all cancer deaths in women are attributable to overweight and obesity [29]. Obesity is associated with increased mortality arising from cancers of the prostate and stomach in men; of the breast (postmenopausal), endometrium, cervix, uterus, and ovaries in women; and of the kidney (renal cell), colon, esophagus (adenocarcinoma), pancreas, gallbladder, and liver in both sexes [29]. However, the relationship between metabolic syndrome and the pathogenesis of cancer is less well established.

Here, we review the association between metabolic syndrome and gastrointestinal cancer, focusing on the pathogenic roles of obesity and adipokines.

## 2. Epidemiology and Epigenetics of Obesity and Metabolic Syndrome

The prevalence of metabolic syndrome, as defined by the 2001 Adult Treatment Panel (ATP) III criteria, was evaluated in 8814 adults in the United States who participated in the third National Health and Nutrition Examination Survey (NHANES III, from 1988 to 1994) [30]. The overall prevalence was 22%, with an age-dependent increase (6.7, 43.5, and 42.0% for the age groups of 20–29, 60–69, and >70 years, resp.).

The prevalence of metabolic syndrome is steadily increasing. Indeed, in NHANES from 1999 to 2002, 34.5% of participants met the ATP III criteria for metabolic syndrome, an increase from 22% in NHANES III (from 1988 to 1994) [30, 31]. In addition, metabolic syndrome, defined by the ATP III criteria revised in 2005, was assessed in 3323 Framingham Heart Study participants, aged 22–81 years who did not have diabetes or CVD at a baseline examination in the early 1990s [32].

39% prevalence in moderately obese and 50% prevalence in severely obese children and adolescents, based on study of 439 obese children and adolescents; metabolic syndrome defined as 3 or more criteria for age and sex.

Epidemiological and animal studies have demonstrated a close link between maternal nutrition and chronic metabolic disease in children and adults [33]. For example, evidence supporting the effects of maternal malnutrition on the health of the offspring was presented in a historical cohort of Dutch individuals whose mothers were exposed to the wartime famine of 1944-1945. Of note, the offspring of women exposed to undernutrition during early pregnancy were more likely to develop metabolic syndrome in adulthood compared with offspring of women who were pregnant either before or after the famine [34].

The current evidence for the fetal influences on later health is mainly attributed to the mother's diet, as maternal obesity, excessive weight gain during pregnancy, and gestational diabetes are known to increase the risk of various diseases in the offspring. Nutritional imbalance (over- or undernutrition) and exposure to environmental chemicals during development can also increase the risk of these diseases, possibly through common pathways [35].

These pathways include DNA methylation, histone modifications, and noncoding RNA expression. Such epigenetic modifications can be passed from one cell generation to the next and, in some cases, if germ cells are targeted, can be transmitted across generations [36].

Disruption of epigenetic mechanisms can result in oxidative stress, obesity, insulin resistance, diabetes, and vascular dysfunction in animals and humans. Hormones, cytokines, and nutritional components can directly activate receptors that stimulate gene expression and can also activate or inhibit the enzymes and pathways that are responsible for DNA methylation, chromatin remodeling, and noncoding RNAs, which ultimately control gene expression. Thus, epigenetic regulatory pathways are the likely targets that mediate the effects of nutrients and environmental toxicants during development and potentially across the entire lifespan.

## 3. Mechanisms

Adipose tissue, particularly visceral fat [37], is an important metabolic tissue that secretes systemic factors that can alter the immunologic, metabolic, and endocrine milieu and promote insulin resistance. Adipose tissue is a metabolically active organ that secretes many adipokines, including TNF, IL-6, and adiponectin. It is also a major source of growth factors, such as IGF-1, which is also produced in the liver. Many of these adipokines can induce insulin resistance syndrome [38].

Within the growth-promoting, proinflammatory environment of the obese state, cross-talk between macrophages, adipocytes, and epithelial cells occurs via obesity-associated hormones, cytokines, and other mediators that may increase the risk of cancer and its progression [39].

### 3.1. Insulin and Insulin-Like Growth Factor

Obesity is strongly associated with insulin resistance, in which the levels of insulin and IGF-1 are elevated. Insulin can act as a mitogen and is associated with several cancers [40]. Insulin is an important growth factor for colonic mucosal cells and colonic carcinoma cells *in vitro* [41–43], while IGF-1 inhibits apoptosis and promotes cell cycle progression, leading to the development of cancer [44, 45].

Most research to date has focused on the systemic mechanisms underpinning the association between obesity and cancer. Potentially relevant pathways include the insulin-IGF axis, sex steroid pathways, and adipocytokine pathways.

Insulin is an anti-inflammatory agent, and anti-inflammatory agents have been shown to reduce the risk of colorectal neoplasms [46, 47]. Therefore, the link between hyperinsulinemia and colorectal neoplasms is unclear.

Obesity is also associated with tissue inflammation, which is at least partly mediated by adipose tissue. It was speculated that subclinical inflammation is associated with the development of colorectal cancer [48] because chronic inflammation can result in colorectal cancer in patients with ulcerative colitis [49].

Therefore, one possible explanation for the link between diabetes and colorectal cancer is hyperinsulinemia. This link was proposed because insulin is an important growth factor for colonic mucosal cells and stimulates the proliferation of colonic tumor cells [41, 42, 50].

The plasma concentrations of IGF-1 and IGF binding protein-3 (IGFBP-3) were reported to influence the risk of colorectal cancer in a prospective cohort of 14,916 men [51]. Subjects with IGF-1 levels in the highest quintile were more likely to develop colorectal cancer compared with subjects with IGF-1 levels in the lowest quintile. By contrast, higher plasma IGFBP-3 levels were protective against colorectal cancer. A similar relationship between serum C-peptide levels (a marker for insulin production) and colorectal cancer risk was reported in the Physicians' Health Study, and this association was independent of IGF-1 and IGFBP-3 levels [52].

Genetic polymorphisms of IGF-1 may also increase the risk of cancer [53]. Polymorphisms of IGF-1 (rs1520220 and rs2195239) were reported to decrease the risk of disease recurrence in Japanese patients with gastric cancer who had undergone curative gastrectomy [54].

An earlier study [55] revealed that metformin, a commonly used oral antihyperglycemic agent belonging to the biguanide family, may reduce the risk of cancer, and improve its prognosis. One explanation for this finding is that metformin reduces the phosphorylation of epidermal growth factor receptor and IGF-1 receptor *in vitro* and *in vivo* [56].

### 3.2. Adipocytokines

Adipocyte-conditioned media can promote tumorigenesis in cancer cells by increasing cell proliferation, invasive potential, angiogenesis, and cross-talk between cancer cells and the surrounding extracellular matrix [45]. These actions are thought to be mediated by adipokines such as adiponectin, leptin, TNF, IL-6, IL-8, IL-10, and IL-1 receptor agonists [57].

Adiponectin is the most abundant adipocytokine and is predominantly secreted from visceral fat adipocytes. Adiponectin levels are inversely correlated with BMI [58] and are generally higher in women than in men. Adiponectin is an insulin sensitizer, with anti-angiogenic and antiinflammatory activities. It can also inhibit tumor growth in animals [59]. Adiponectin levels are also inversely correlated with the risk of gastric cancer [60]. Similarly, it may protect against liver tumorigenesis, as reduced adiponectin expression is associated with poor prognosis in obese patients with hepatocellular carcinoma (HCC) [61]. Additionally, visceral fat accumulation and decreased plasma adiponectin levels were associated with the development of colorectal adenoma in humans [62].

Mice with disruptions in the adiponectin gene are more likely to develop intestinal tumors, with decreased activation (i.e., phosphorylation) of AMP-activated protein kinase and increased PAI-1 levels compared with wild-type mice.

Several different molecular weight isoforms (i.e., high, medium, and low molecular weight adiponectin) can be detected in the vascular system. The biological effects of these isoforms are mainly mediated through two classical adiponectin receptor subtypes: AdipoR1 and AdipoR2 [63]. Several studies have examined the role of adiponectin receptors in the pathogenesis of colon cancer, yielding controversial results. *In vitro*, adiponectin suppressed colon cancer cell proliferation via AdipoR1- and AdipoR2-mediated AMPK activation [64]. In humans, the expression of adiponectin receptors was reported to be significantly higher [65] in areas occupied by colorectal tumors or similar [66] to that of normal colorectal tissue in individuals with colorectal cancer.

Leptin is an adipocyte-specific hormone that acts centrally to regulate appetite and bodyweight [67] and therefore acts as a signal for sufficient energy intake. Higher leptin levels are correlated with adiposity and are associated with colon cancer [68–70]. *In vitro* studies have confirmed that leptin promotes cell proliferation, angiogenesis, and metalloproteinase expression in esophageal and colon cancer cell lines [71].

Resistin, a 12.5-kDa protein encoded by a region on chromosome 19 in humans, is a 108 amino acid prepeptide [72]. In humans, resistin is mainly produced by peripheral blood mononuclear cells, macrophages, bone marrow, pancreatic cells, and adipocytes, as well as the spleen and muscles [73]. In the context of inflammation, resistin induces the expression and release of IL-1*β*, IL-6, IL-8, IL-12, TNF, and Toll-like-receptor-2 through the nuclear factor-*κ*B pathway [74]. Resistin is also thought to mediate insulin resistance, although this effect appears to be limited to animals [74]. In humans, circulating resistin levels were greater in patients with colon cancer [75, 76] or colon adenoma [75] compared with control subjects. Resistin levels were not dependent on cancer stage, grade of dysplasia, or location [75]. In molecular-based studies, the resistin-related SNP C-420G genotype was associated with an increased risk of colorectal cancer (per allele odds ratio [OR] 1.18, 95% confidence interval [CI] 0.99–1.40) [134], whereas the resistin-promoter SNP C–180G was not associated with elevated resistin levels in patients with colon cancer [135].

### 3.3. Immunomodulation

Excess adipose tissue is associated with chronic, systemic, low-grade inflammation [77]. The amount of adipocytokines produced by adipose tissue is strongly influenced by the immune cells present in adipose tissue [78–80]. Adipose tissue in obese people shows extensive macrophage infiltration, the number of which is correlated with the level of adiposity [81].

The immune system plays a fundamental role in antitumor immunity, and, under certain circumstances, it can promote tumor development and progression. In most studies, the density of tumor-associated macrophages was correlated with increased angiogenesis, tumor invasion, and poor prognosis [82].

## 4. Metabolic Syndrome and Gastrointestinal Cancer

### 4.1. Barrett's Esophagus and Esophageal Adenocarcinoma

Gastroesophageal reflux disease (GERD) is a common cause of morbidity and healthcare use in many countries. A positive association between increased BMI and the presence of GERD was reported in the United States, but this relationship became apparent only after stratification by country and the level of BMI [83].

Overweight and high BMI are associated with an increased risk of esophageal adenocarcinoma and adenocarcinoma of the gastric cardia, even in subjects with a normal BMI [84–86]. In a meta-analysis of eight studies, the pooled adjusted ORs for esophageal adenocarcinoma among subjects with a BMI of 25–30 kg/m^2^ or >30 kg/m^2^ were 1.52 and 2.78, respectively [84].

Adipose tissues, particularly metabolically active visceral adipose tissues, produce large amounts of adipocytokines, including IL-6 and TNF [87], which may play a role in GERD or subsequent carcinogenesis. Leptin is predominantly secreted from adipose tissues, and its serum levels increase in proportion to body fat mass [88]. Leptin was reported to stimulate cell proliferation and inhibit apoptosis in Barrett's-derived esophageal adenocarcinoma cells [89].

Obesity is also associated with upregulated leptin receptor and adiponectin receptor expression in esophageal adenocarcinoma cells. The association between leptin receptor and adiponectin receptor expression with tumor stage suggests that adipocytokines may influence tumor biology. Over 90% of esophageal adenocarcinomas were reported to express the leptin receptor, with most (67%) showing markedly upregulated expression [90].

The systemic inflammatory state that develops as a consequence of disturbed metabolism in obese patients and the associated impact of adipocytokines and procoagulants released by adipocytes in visceral fat may underlie the relationship between obesity and esophageal adenocarcinoma [91].

Interestingly, subjects with serum insulin and IGF-1 levels in the highest tertiles had the highest risk of Barrett's esophagus. Serum IGF-1 levels in the highest tertile were associated with an increased risk of Barrett's esophagus (adjusted OR 4.05, 95% CI 2.01–8.17) compared with a control group who underwent screening colonoscopy, but were not significantly different from a control group of subjects with GERD [92].

### 4.2. Gastric Adenocarcinoma

Excess body weight and obesity are associated with an increased risk of gastric cancer [93]. For example, in a meta-analysis of cohort studies involving 9492 patients with gastric cancer, excess body weight (BMI 25–30 kg/m^2^) and obesity (BMI > 25 kg/m^2^) were associated with an increased risk of cancer of the gastric cardia among Asians and non-Asians, and with gastric cancer among non-Asians. The strength of these associations increased with increasing BMI. However, overweight was not an independent prognostic factor for long-term survival in Western patients with gastric cancer [94].

### 4.3. Colorectal Adenocarcinoma

 Obesity is an important risk factor for colorectal cancer. Two large prospective cohort studies have shown that obesity increases the risk of colorectal cancer by approximately 1.5-fold compared with normal weight individuals (BMI 18.5–24.9 kg/m^2^) [95, 96]. Similarly, in a systematic review of data from 29 studies involving 37,334 patients, each 5 kg/m^2^ increase in BMI was associated with a 24% increased incidence of colon and rectal cancer in men, and a 9% higher incidence of colon cancer in women [97]. 

Many studies have demonstrated that the association between obesity and colorectal cancer is stronger in men than in women. This was hypothesized to be due to differences in adipose tissue distribution, as the more pronounced visceral adiposity in men than in women mirrors the apparent difference in risk [98].

Colorectal cancer and adenoma disease are associated with dietary factors, such as red meat, high fat content, and inadequate fiber intake. Of note, high intake of red meat and processed meat was associated with increased risk of colorectal, colon, and rectal cancers [99, 100].

Two studies also found an increased risk of colorectal cancer in subjects with metabolic syndrome [25, 101]. Interestingly, in one of these studies, the risk was higher in men than in women [25].

It is also becoming apparent that adiposity is the factor that is most strongly associated with the risk of colorectal cancer [102]. Central obesity, triglyceride levels, and metabolic syndrome are all risk factors for colorectal adenoma, including advanced adenoma and multiplicity [103]. Metabolic syndrome was also associated with an increased risk of adenoma. This association between multiple syndrome and colorectal adenoma was observed regardless of advanced/low-risk adenoma and multiplicity [103].

### 4.4. HCC

Several epidemiologic studies have revealed a possible link between diabetes mellitus and HCC [17, 69, 104, 105]. However, the associations between diabetes and HCC should be interpreted with care. In many patients, glucose intolerance is secondary to the development of cirrhosis. Therefore, diabetes may be a surrogate for cirrhosis, which increases the risk of HCC. Many patients with diabetes also have nonalcoholic fatty liver disease (NAFLD), another risk factor for HCC. There is growing evidence that NAFLD represents an increasingly common underlying liver disease in patients with HCC [28, 106, 107]. It is likely that NAFLD causes HCC via cirrhosis, although the exact pathogenesis is unclear. Nevertheless, one study showed that HCC in patients with nonalcoholic steatohepatitis was associated with obesity, diabetes, hypertension, and male sex [107]. Unfortunately, very few studies have specifically examined the association between metabolic syndrome and cancer risk. The predominant manifestation of metabolic syndrome in the liver is NAFLD, which can progress to cirrhosis and ultimately HCC [108]. Interestingly, treatment with a thiazolidinedione or metformin was associated with a decreased risk of HCC in patients with diabetes [69].

### 4.5. Gallbladder and Biliary Carcinoma

An association between diabetes mellitus and cancer of the biliary tract was reported in several case-control and cohort studies. In a meta-analysis of 15 studies, patients with diabetes had a significantly higher risk of cholangiocarcinoma relative to individuals without diabetes [109]. A study of 743 patients with intrahepatic cholangiocarcinoma found that the presence of metabolic syndrome (defined by the presence of three of the following: elevated waist circumference/central obesity, dyslipidemia, hypertension, or impaired fasting glucose) was a risk factor for intrahepatic cholangiocarcinoma [110]. Therefore, it is unclear whether diabetes itself or other associated conditions (e.g., obesity and hyperlipidemia) are the major risk factors for cholangiocarcinoma.

### 4.6. Pancreatic Adenocarcinoma

Several studies have revealed a link between high body mass, lack of physical activity, and pancreatic cancer risk [111–117]. A BMI of ≥30 kg/m^2^ was associated with a significantly increased risk of pancreatic cancer compared with a BMI of <23 kg/m^2^. Height was also associated with an increased risk of pancreatic cancer. On the other hand, an inverse association was observed for moderate physical activity when comparing the highest and lowest categories, particularly among individuals with a BMI of ≥25 kg/m^2^. It was also reported that high BMI is associated with decreased survival in patients with pancreatic cancer, although the mechanism for this association was not determined [118].

Other researchers have suggested that overweight and obese individuals develop pancreatic cancer at a younger age than do patients with a normal weight, and the rate and duration of survival are lower, once pancreatic cancer is diagnosed [115].

## 5. Conclusions

Metabolic syndrome represents a cluster of metabolic abnormalities and is defined as the presence of three or more of the following factors: abdominal obesity (i.e., increased waist circumference), elevated triglycerides, low HDL cholesterol, high blood pressure, and high fasting glucose. Multiple hormones, growth factors, cytokines, and other mediators associated with the obese state and metabolic syndrome enable cross-talk between macrophages, adipocytes, endothelial cells, and epithelial cells and therefore contribute to cancer-related processes. Efforts to increase our knowledge of the underlying mechanisms are crucial to help us design effective therapeutic strategies to prevent and treat metabolic abnormalities.

The association of gastrointestinal diseases with metabolic syndrome is summarized in Table 2. The incidence of cancer is clearly increased in obese individuals, but it is not yet clear whether weight loss can decrease the incidence of cancer [119].

Recent studies have shown that the risk of disease may be induced very early in the life course and it may be modifiable by nutrients. Consequently, it seems rational to improve nutrition and reduce exposure to environmental chemicals before and during pregnancy, and during the first few years of life. Such changes are likely to have a significant impact on reducing the disease burden and the associated healthcare costs.

## Figures and Tables

**Table 1:** Definitions of metabolic syndrome.

	WHO [3]	IDF [23]	AHA/NHLBI [21]	ATP III [5]	Japanese consensus [22]
	Impaired glucose regulation or hyperinsulinemia and more than two factors	WC and two or more risk factors	at least 3 of	Three or more of:	WC and two or more risk factors
Abdominal obesity	Waist: hip ratio ≥ 0.85 or BMI ≥30 kg/m^2^	waist circumference at least 94 cm for Europoid men or 80 cm for Europoid women 90 cm for South Asian and South-East Asian men or 80 cm for South Asian and South-East Asian women 85 cm for Japanese men or 90 cm for Japanese women	WC (102 cm (40 inches) or higher in men 88 cm (35 inches) or higher in women)	Men: WC ≥ 102 cm Women: WC ≥88 cm	Men: WC ≥ 85 cm Women: WC ≥90 cm
Impaired glucose regulation	Fasting plasma ≥ 6.1 mmol/L or 2 h postglucose load ≥ 7.8 mmol/L	Fasting plasma ≥ 100 mg/dL (5.6 mmol/L) or previously diagnosed type 2 DM	Fasting plasma ≥ 100 mg/dL (5.6 mmol/L) or previously diagnosed type 2 DM	110–126 mg/dL (6.1–7.0 mmol/L)	Fasting plasma ≥ 110 mg/dL
Hyperinsulinemia	Fasting serum insulin > 1/3rd quartile for control group	Not included	Not included	Not included	Not included
Triglycerides	≥150 mg/dL (1.7 mmol/L)	≥150 mg/dL (1.7 mmol/L)	≥150 mg/dL (1.7 mmol/L)	≥150 mg/dL (1.7 mmol/L)	≥150 mg/dL (1.7 mmol/L) or treatment for same
HDL concentration	Not included	Men: <40 mg/dL (1.03 mmol/L) Women: <50 mg/dL (1.3 mmol/L)	Men: <40 mg/dL (1.03 mmol/L) Women: <50 mg/dL (1.29 mmol/L)	Men: <40 mg/dL (1.03 mmol/L) Women: <50 mg/dL (1.3 mmol/L)	<40 mg/dL (1.03 mmol/L)
Hypertension	≥140/90 mmHg	≥130/≥85 mmHg or treatment for same	elevated blood pressure 130/85 mmHg or higher or on medication	≥130/≥80 mm Hg	≥130/≥85 mm Hg or treatment for same
Microalbuminuria	Albumin/creatinine ratio > 30 mg/g	Not included	Not included	Not included	Not included

WHO: World Health Organization; IDF: International Diabetes Federation; NHLBI: National Heart, Lung and Blood Institute; AHA: American Heart Association; World Heart Federation; ATP III: Adult Treatment Panel III, report of the National Cholesterol Education; WC: waist circumference; BMI: body mass index; DM: diabetes mellitus; HDL: high-density lipoprotein.

**Table 2:** Metabolic syndrome and gastrointestinal disease.

	Reference	Year	Patients no.	Definitions of metabolic syndrome	Relative risk (RR) or odds ratios (OR)	*P* value
Colorectal cancer	Pelucchi et al. [24]	2010	1378	IDF criteria	Men: OR, 2.09 (95% CI, 1.38–3.18)	<0.001
				>3 components	Women: OR, 1.15 (95% CI, 0.68–1.94)	*P* = 0.22
	Ahmed et al. [25]	2006	194	ATP III	Men: RR, 1.78 ( 95% CI, 1.0–3.6)	
				>3 components or more	Women: RR, 1.16 ( 95% CI, 0.6–2.2)	
Nonalcholic fatty liver disease	Hamaguchi et al. [26]	2005	816	Presence of the metabolic syndrome	Men: OR, 4.00 (95% CI, 2.63–6.08)	<0.001
				ATP III	Women: OR, 11.20 (95% CI, 4.85–25.87)	<0.001
liver fibrosis	Marchesini et al. [27]	2003	304	ATP III	OR, 3.5 (95% CI, 1.1–11.2)	*P* = 0.032
NASH	Marchesini et al. [27]	2003	304	ATP III	OR, 3.2 (95% CI, 1.2–8.9)	*P* = 0.26
Hepatocellular carcinoma (HCC)	Welzel et al. [28]	2011	3649	ATP III	OR, 2.13; (95% CI = 1.96–2.31)	*P* < 0.0001
Intrahepatic cholangiocarcinoma	Welzel et al. [28]	2011	743	ATP III	OR, 1.56; (95% CI = 1.32–1.83)	*P* < 0.0001
